# Postprandial Hypotension in Clinical Geriatric Patients and Healthy Elderly: Prevalence Related to Patient Selection and Diagnostic Criteria

**DOI:** 10.4061/2010/243752

**Published:** 2010-09-30

**Authors:** Narender P. Van Orshoven, Paul A. F. Jansen, Irène Oudejans, Yvonne Schoon, P. Liam Oey

**Affiliations:** ^1^Department of Neurology, Rudolf Magnus Institute of Neuroscience, University Medical Center Utrecht, P.O. Box 85500, 3508 GA Utrecht, The Netherlands; ^2^Department of Geriatric Medicine, University Medical Center Utrecht, P.O. Box 85500, 3508 GA Utrecht, The Netherlands; ^3^Department of Geriatric Medicine, Elkerliek Hospital, Helmond, The Netherlands

## Abstract

The aims of this study were to find out whether Postprandial hypotension (PPH) occurs more frequently in patients admitted to a geriatric ward than in healthy elderly individuals, what the optimal interval between blood pressure measurements is in order to diagnose PPH and how often it is associated with symptoms.The result of this study indicates that PPH is present in a high number of frail elderly, but also in a few healthy older persons. Measuring blood pressure at least every 10 minutes for 60 minutes after breakfast will adequately diagnose PPH, defined as >20 mmHg systolic fall, in most patients. However with definition of PPH as >30 mmHg systolic fall, measuring blood pressure every 10 minutes will miss PPH in one of three patients. With the latter definition of PPH the presence of postprandial complaints is not associated with the existence of PPH.

## 1. Introduction

Postprandial hypotension (PPH) was defined in the past as a fall in systolic blood pressure of ≥20 mmHg within 2 hours of eating a meal, comparable with the definition of postural hypotension [1]. A recent study using sphygmomanometer readings has shown that in subjects with hypertension the cutoff should be a fall of 30 mmHg and not 20 mmHg in systolic pressure [2]. Potential symptoms of a fall in blood pressure include dizziness, syncope, and falls. It has been described frequently in elderly individuals, with a higher incidence in certain risk groups, namely, in 24%–33% of elderly residents of nursing homes [3], in almost 50% of elderly patients with unexplained syncope [4] and in 67% of hospitalized geriatric patients [5]. Risk groups are patients with autonomic dysfunction in diabetes mellitus [6], hypertension [7, 8], Alzheimer's disease [9] and Parkinson's disease [10, 11] although PPH has been reported to occur in 33% of healthy individuals [12]. In the long term, PPH is associated with an increased incidence of falls, syncope, new coronary events, new stroke, and higher total mortality [13]. People who have PPH are at risk of developing cerebral ischemia [14].

The pathophysiology of PPH is probably multifactorial, possibly involving an attenuated baroreflex, an attenuated reflex increase in sympathetic activity by activation of stretch receptors in the stomach (gastrovascular reflex) [15], sympathetic dysfunction (e.g., autonomic neuropathy in diabetes mellitus, Parkinson's disease), and patients with an incapability to increase cardiac output due to heart failure or any combination of these factors. Thus, PPH is likely to occur in frail elderly with extensive comorbidity. There is no consensus on the test conditions that should be used to diagnose PPH. The characteristics of the actual decrease in blood pressure in PPH are not clearly defined: when does blood pressure start to decrease after a meal, how long does the decrease last, and what is the magnitude of the decrease in blood pressure? It is not clear what time interval and which method of blood pressure measurement should be used. In most studies, blood pressure was measured with a sphygmomanometer at intervals varying from 3 up to 60 minutes (Table 1) [3, 5, 16–21]. If the frequency of measurements is too high, participants are likely to experience discomfort. Some authors used continuous blood pressure measurements (finger arterial blood pressure) [11, 22, 23], which might be uncomfortable during prolonged measurements. In most studies standardized test meals were used.

The aims of this study were to establish the prevalence, duration, and association with symptoms of PPH in patients admitted to a geriatric ward, compared with healthy elderly individuals. Patients were provided with a continental breakfast. We measured beat-to-beat blood pressure and tested different intervals between blood pressure measurements to diagnose postprandial hypotension adequately.

## 2. Methods

### 2.1. Subjects

Consecutive patients admitted to the geriatric ward of the University Medical Center Utrecht over 6 months were eligible for inclusion. This ward serves elderly patients with a wide range of acute and chronic diseases and referrals for diagnostic work-up or therapy. Most patients are referred by general practitioners or geriatricians. Inclusion criteria were being able to give informed consent and to walk (with or without walking aid). Exclusion criteria were myocardial infarction less than 3 months earlier, any acute illness, uncontrolled metabolic disease, resting systolic blood pressure more than 200 mmHg, cardiovascular disease (aortic stenosis, intermittent claudication, and angina pectoris), dysphagia, life expectancy less than 3 months, and use of medication that affects blood pressure and which could not be discontinued for 24 hours. All subjects were studied in the week prior to discharge. All participants gave written informed consent prior to the examination. Healthy elderly individuals, recruited by advertisement in a weekly newspaper, were screened by telephone and underwent a physical examination before being matched for age with the geriatric patients. All subjects were healthy according to modified exclusion criteria defining medically stable elderly subjects for exercise studies [24]. Subjects who were taking medications that affect blood pressure that could not be withdrawn for at least 24 hours were excluded as well. All subjects gave written informed consent prior to the study. The measurements were carried out in the same period as in the patient group.

### 2.2. Instrumentation

Blood pressure was recorded using Portapres (TNO-TPD Biomedical Instrumentation, Netherlands Organization for Applied Scientific Research). Portapres is a non-invasive method to record beat-to-beat blood pressure alternately from two adjacent fingers and is accurate for measuring changes over time, but not for measuring absolute values [25]. The method is based on the volume-clamp method of Peñáz and the Physiocal criteria of Wesseling [26, 27]. It also measures heart rate and hydrostatic height of the hand (to correct for pressure changes due to vertical heart-hand distance changes). Beatscope software was used to analyze the measurements (Beatscope 1.0; TNO-TPD Biomedical Instrumentation Netherlands Organization for Applied Scientific Research).

### 2.3. Study Protocol

Twenty-four hours before the measurements, all medication that affects blood pressure was withdrawn. After an overnight fast, the subjects were allowed to take their regular medication (with the above exception) with some water 2 hours before breakfast. They could select the ingredients of their continental breakfast, to mimic normal conditions as closely as possible, but were offered weak tea, because caffeine in coffee might affect blood pressure [28]. After breakfast, subjects were not allowed to eat or drink until measurements were completed. Blood pressure was measured after a supine period of 15 minutes and from 15 minutes before until 135 minutes after the start of breakfast, with subjects in sitting position. To avoid vasoconstriction-related inconsistency in measurements, the hand connected to the Portapres device was placed on a warm cherry stone pillow [29]. Symptoms or complaints associated with PPH (light-headedness, dizziness, tiredness, hazy vision) were scored every 15 minutes on a 3-point scale (absent, moderate, or severe). The total intake of calories, carbohydrates, and fat was calculated afterwards. The study protocol was approved by the Medical, Ethical Testing Committee of the University Medical Center Utrecht.

### 2.4. Data Analysis

Differences between the groups at baseline and maximum changes from baseline were determined with a Mann-Whitney *U* test. The chi-square and Fisher exact tests were used for comparison of the presence of PPH with PPH-related symptoms. 

In this study, PPH was defined as a decline in systolic blood pressure of at least 20 mmHg, determined by calculating the difference between the minimum systolic blood pressure values before and within 120 minutes after the start of breakfast. According to the results of a recent study [2], we also analyzed the presence of PPH defined as a fall in systolic blood pressure of 30 mmHg or more.

Because we were not interested in beat-to-beat blood pressure variations, we calculated mean values over 2-minute periods. The start of PPH and the time of maximal fall in blood pressure are expressed in minutes after the completion of breakfast. To determine the minimum interval between blood pressure measurements for diagnosis of PPH, the maximum decrease in blood pressure was calculated for different intervals between measurements, which was done by omitting 1 or more of the 2-minute blood pressure measurements from calculations. The number of patients diagnosed with PPH was calculated, using these different intervals.

Differences were considered significant with a *P* < .05. Data are expressed as means ± standard deviation (SD) unless otherwise specified.

## 3. Results

### 3.1. Patient Characteristics

During the study period, 101 patients were admitted to the geriatric ward, 68 of whom were excluded because they met one or more of the exclusion criteria (in most cases because they suffered form an acute illness). Five patients did not give consent, and five others could not participate because of technical difficulties. The remaining 22 patients (32% male) were studied. They were admitted because of metabolic disorders (4 patients), mobility disorders (4), anemia (3), Parkinsonism (3), delirium (2), syncope (2), or miscellaneous (4). Mean age was 84 years (SD ± 5, range 74–93). Resting blood pressure was 140/65 mm Hg (SD ± 27/15, range 104–200/31–90) and heart rate 67/min (SD ± 10, range 40–80). Of these patients, 45% had a history of cardiovascular disease (other than hypertension), 32% had a history of hypertension (partially overlapping the group 45% of subjects with cardiovascular disease), 27% had Parkinson's disease (all were Hoehn and Yahr stage 2 to 3), 18% had a history of cerebrovascular disease, and 14% had diabetes mellitus.

Of the 53 healthy elderly subjects who responded to the advertisement, 20 were excluded because they met one or more of the exclusion criteria, 2 did not give consent after reading the patient information, and 11 were excluded because of age mismatching. The remaining 20 healthy elderly were compared with the 22 geriatric patients. There were no differences in baseline characteristics between the two groups (Table 2). Of the healthy elderly individuals, 23% had with hypertension; other diseases were not present.

### 3.2. Postprandial Hypotension in Frail and Healthy Elderly

**Figure 1:**
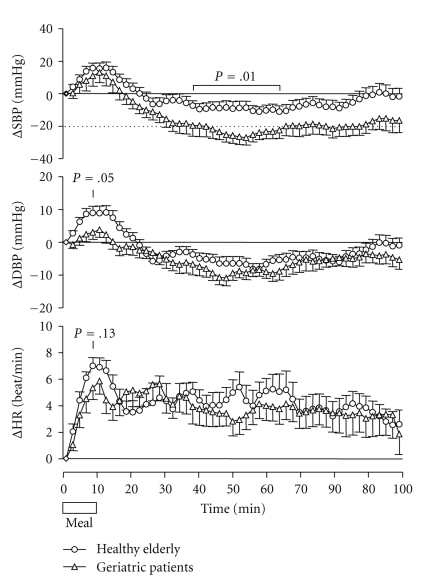
Mean change in systolic blood pressure (SBP), diastolic blood pressure (DBP), and heart rate (HR) in 22 geriatric patients and 20 healthy elderly individuals during and after breakfast (*P*-values of differences between groups).

Figure 1 shows mean systolic and diastolic blood pressure, and heart rate during and after breakfast of individuals. There were no significant differences between the two groups, and blood pressure and heart rate increased in both groups during the meal. The increase in diastolic blood pressure and heart rate tended to be higher in the healthy elderly group (*P* = .05 and *P* = .13, resp.). After the meal, systolic and diastolic blood pressure decreased below baseline values in both groups, but the fall in systolic blood pressure was significantly greater in the patient group (mean values over period 40–60 minutes after the start of breakfast: 24.1 ± 19.6 versus 9.6 ± 15.8 mmHg; *P* = .01). Thus PPH, defined as a systolic fall of 20 mmHg or more, occurred in 20 (91%) of 22 patients and in 8 of 20 (40%) healthy elderly individuals (*P* = .001, MWU test). PPH defined as a fall of 30 mmHg or more was present in 10 (45%) patients and three (15%) healthy persons (*P* < .001). The characteristics of the blood pressure fall in the subjects with PPH are shown in Table 3. There were no statistically significant differences between the 2 groups in the moment of onset and duration of PPH, maximum fall in systolic blood pressure, time between end of breakfast and maximum fall in systolic blood pressure, and increase in heart rate, defined as the difference between maximum heart rate in the baseline and in the postprandial period.

### 3.3. Minimal Required Interval for Blood Pressure Measurements

**Figure 2:**
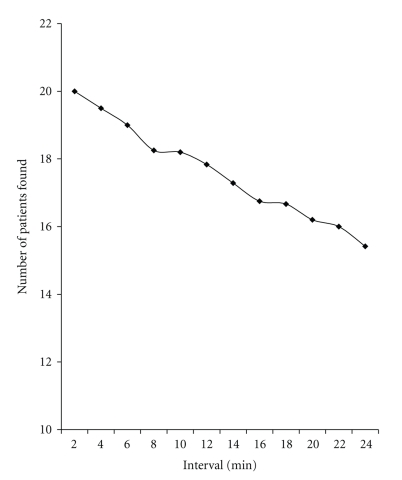
Time interval of blood pressure beat-to-beat measurements related to the detection of postprandial hypotension in 22 geriatric patients.

We determined the interval between blood pressure measurements that was most appropriate for detecting PPH in the individuals with PPH in the group of patients that were admitted to the geriatric ward. PPH lasted 2 minutes in two patients, 6 minutes in one patient, and 8 minutes in two patients. It lasted between 14 and 108 minutes in the remaining 15 patients (see Figure 2). Eight patients had two periods of PPH. If one of every two 2-minutes values was left out, a maximum of one patient would be missed, depending on which of the first 2-minute values was left out. If blood pressure was measured at 4-minute intervals (one of three 2-minute measurements omitted), at least one patient would be missed. If blood pressure was measured at 6-minute intervals (one of four 2-minute measurements omitted), at least 2 patients (10%) would be missed; with intervals of 10 and 15 minutes, 3 (15%) and 4 (20%) of the patients with PPH would not be detected (Figure 2). 

### 3.4. Association of Presence of PPH and Complaints

Of the 42 included subjects, 18 developed PPH-related symptoms and 24 had no complaints (Table 4). Seventeen of the 18 subjects (95%) who developed PPH-related symptoms, had a simultaneous decrease in blood pressure greater than 20 mmHg, consistent with PPH. Thirteen of the 24 subjects (54%) who did not have PPH-related symptoms did not have a significant decrease in blood pressure. PPH-related complaints were present in 7 (70%) patients with PPH and none of the healthy persons with PPH with a systolic fall of >30 mmHg. To calculate the sensitivity and specificity of PPH-related symptoms for the diagnosis of PPH, we considered patients to be symptomatic if at least one of the symptoms was scored as “moderate” during the test. The sensitivity of PPH-related symptoms for a significant decrease in blood pressure was 17/28 = 0.61, the specificity was 13/14 = 0.93, the positive predictive value was 17/18 = 0.94, and the negative predictive value was 13/24 = 0.54. The presence of PPH-related symptoms was strongly associated with a decrease in blood pressure greater than 20 mmHg (*P* < .001). However with PPH defined as a systolic fall of >30 mmHg, an association could not be found. The sensitivity of PPH-related symptoms for a significant decrease in blood pressure was 7/13 = 0.53, the specificity was 18/29 = 0.62, the positive predictive value was 7/18 = 0.39, and the negative predictive value was 18/24 = 0.75. Also an association between supine systolic hypertension >160 mmHg in rest and PPH could not be found. Only four patients and one healthy subject had a supine blood pressure >160 mmHg after 15 minutes rest; three of them had PPH.

## 4. Discussion

We found PPH to be present in a high number of frail elderly patients, but also in a considerable number of healthy elderly. The prevalence of PPH we found in elderly patients (91%) is higher than previously reported [3, 7, 30]. Recently a study suggested that the cutoff of orthostatic fall in the elderly should be 30 mmHg in systolic pressure or more and not 20 mmHg [2]. Using this cutoff value, the prevalence of PPH in our study is 45% in the patient group and 15% in the healthy group. The higher prevalence in the patient group is probably inherent on the comorbidity of this group. There was considerable variability in the time of onset, duration, and maximum decrease in blood pressure in the subjects with PPH, which supports the hypothesis that PPH is not caused by the failure of a single mechanism, but rather by the failure of several, and that there is considerable variation between subjects.

Despite this variability, PPH started within an hour of eating breakfast in 18 of 20 patients (90%). Assuming that changes in systolic blood pressure measured with a sphygmomanometric device are similar to the 2-minute mean Portapres values that were used in this study, blood pressure measured at 6-minute intervals would have identified at least 18 patients with PPH (90%); measurement with 10-minute intervals would have identified at least 17 patients (85%) with PPH (Figure 2). However with definition of PPH as >30 mmHg systolic fall, measuring blood pressure every 6 minutes will miss PPH in one of four and with 10 minutes interval in one of three patients.

Beat-to-beat measurements will increase the number of short-lasting falls in postprandial blood pressure detected relative to the number detected when blood pressure is measured with a sphygmomanometer. However, in general practice, this method is not attractive because continuous blood pressure measurements are comprehensive, require skill to get reliable results, and require relatively expensive equipment. For this reason, automatic sphygmomanometer measurements, with devices such as Spacelab, are preferred (Table 1).

In this study, the subjects ate a continental breakfast, in which the release of glucose is quite constant whereas other studies used standardized liquid meals, in which the release of glucose is rapid. We chose a standardized continental breakfast as being more representative of the normal situation. Moreover, we hypothesize that differences in the severity of PPH observed with high and low carbohydrate-containing meals can be explained by differences in the rate of glucose uptake rather than by differences in the total number of calories consumed, a hypothesis that is supported by the findings of O'Donovan et al. [16]. They found that the fall in systolic blood pressure was greater with a higher rate of glucose delivery into the duodenum. Despite the probably relatively low rate of glucose delivery with our continental breakfast, a large proportion of our subjects developed PPH.

Our study had some potential limitations. We chose to withdraw medication that affects blood pressure for 24 hours to eliminate transient effects of medication that could occur directly after ingestion. However, we cannot rule out the possibility that these medications affect the mechanism of PPH. We calculated mean values of blood pressure over 2-minute periods and may thus have missed a short-lasting fall (less than 2 minutes) in blood pressure. However, such a short-lasting fall in blood pressure may not be of clinical relevance. It was our goal to mimic the normal physiological condition by using a continental breakfast instead of standardized meal and to allow subjects to choose their breakfast. Because subjects were not active after they finished their breakfast, we cannot rule out that the fall in blood pressure was the effect of drowsiness or even sleep as might occur during the period of inactivity after the meal, adding to the occurring hypotension, although a researcher was constantly in the room and a symptom-questionnaire was evaluated every 15 minutes, limiting the presence of drowsiness and its potential influence. There is currently no standardized clinically meaningful definition of PPH [31].

In the current study, it is surprising that blood pressure fall up to more than 30 mm Hg is still well tolerated in six subjects (3 patients and 3 healthy subjects) without any symptoms. 

The mechanism(s) mediating the effects of meal on the hypotensive response remain uncertain, and there are a number of possibilities which warrant further exploration. Symptoms due to reduced cerebral perfusion can be manifested as dizziness, light-headedness, weakness, dyspnea, sweating, or syncope. Cerebral hypoperfusion develops when cerebral autoregulation fails. The autoregulated range is known, typically 70–140 mmHg. Cerebral autoregulation is ineffective when the blood pressure falls to <70 mmHg, and here the cerebral perfusion might be severely compromised. However, patients with chronic orthostatic hypotension may have an expansion of their autoregulated range to lower BP [32]. Such patients and perhaps elderly patients need much greater fall in blood pressure than 30 mmHg.

As neurogenic orthostatic hypotension, severely afflicted patients are unable to stand without experiencing symptoms. We cannot, however, exclude the possibility that there is some kind of autonomic dysfunction in this very elderly group. Aging, leading to abnormalities of parasympathetic and sympathetic control of heart rate and blood pressure, may be associated with concomitant autonomic afferent sensory fiber neuropathy. Degeneration or dysfunction of these afferent fibers may result in interruption of pain or other autonomic disorders sensations [33]. Reduced appreciation for ischemic events such as pain or dizziness can impair timely recognition of ischemia or infarction, thereby delaying appropriate therapy. Hence, patients who have no symptoms of this hypotension could be jeopardized because the longer threshold permits them to continue the event (meal) despite increasing ischemia. In this study, we did not search for signs of cardiovascular pathology, and we did not follow our patients and the healthy subjects up. Therefore we do not know the prognostic significance of PPH in this study groups.

From the clinical perspective, it is important to measure blood pressure following a meal in patients who have unexplained syncope and whose orthostatic stress test result is normal. Old age is associated with diminished homeostatic regulation of many physiological functions. Reduced fluid and food intake and common medical conditions in the elderly, such as gastrointestinal disease (malabsorption syndromes), can cause dehydration. Dehydration and the use of diuretics or other agents may reduce the cardiac output in the elderly. In subjects with PPH, these factors might induce severe hypotension, and this is a potential trigger for ischemic stroke or acute myocardial infarction.

In conclusion, PPH occurs in a high number of frail elderly patients admitted to a geriatric ward. PPH was also present in a few relatively healthy elderly. Measuring blood pressure every 10 minutes, starting from 15 minutes before until 60 minutes after the end of a regular breakfast, using a sphygmomanometer, will detect about 70% of geriatric patients with PPH. This seems a patient friendly, practical, and adequate way to evaluate PPH in clinical practice. The presence of postprandial complaints during the measurement is not a good indicator of the existence of PPH.

## Figures and Tables

**Table 1:** Methods used to measure postprandial hypotension.

Author (reference)	Subjects (*n*)	Studygroup	Equipment	Time-interval (min.)	Duration measurement (min.)
Aronow and Ahn 1994 [3]	499	Long-term health care facility	Mercury sphygmomanometer	15	120
Jansen et al. 1996 [17]	22	Nursing home patients	Dinamap	5	90
Kohara et al. 1998 [20]	121	Hospitalized hypertensive patients	ABPM	30	24 hour
Puisieux et al. 2000 [18]	120 versus 36	In patients with syncope or falls versus control patients	Spacelab	15	24 hour
O'Donovan et al. 2002 [16]	8	Healthy elderly	Not mentioned	3	120
Kawaguchi et al. 2002 [19]	20 versus 20	Healthy elderly versus healthy Young individuals	Mercury sphygmomanometer	30	120
Vloet et al. 2005 [5]	58	Geriatric patients	Spacelab	10	90
Fisher et al. 2005 [21]	179	Long-term health care facility	Spacelab and mercury sphygmomanometer	60	60

ABPM: ambulatory blood pressure measurement.

Duration measurement: total time measured after finishing breakfast.

**Table 2:** Baseline characteristics of patients admitted to a geriatric ward and of healthy elderly individuals.

	Patients (*n* = 22)	Healthy elderly (*n* = 20)	*P*
Male versus Female (*n*)	7 versus 15	2 versus 18	.14
Age (yr)	84 ± 5 (74–93)	82 ± 4 (75–88)	.07
Quetelet index (kg/m^2^)	27 ± 7 (18–42)	26 ± 3 (22–33)	.89
Calorie intake (kcal)	345 ± 118 (185–603)	306 ± 52 (191–367)	.52
Carbohydrate intake (g)	38 ± 12 (20–70)	35 ± 9 (19–46)	.67
Fat intake (g)*	16 ± 7 (4–27)	12 ± 3 (7–17)	.05
Systolic BP (mmHg)**	140 ± 27 (104–204)	136 ± 20 (94–164)	.48
Diastolic BP (mmHg)**	65 ± 15 (31–90)	65 ± 11 (47–80)	.99
Heart rate (bpm)**	67 ± 10 (40–80)	68 ± 10 (42–80)	.53
Vascular resistance (MU)**	1.9 ± 1.4 (0.3–5.1)	2.0 ± 1.8 (0.7–9.1)	.94

Mean ± standard deviation (range).

*Intake during test meal.

**Minimum values in 15 minutes before breakfast.

*P*-values calculated with Mann-Whithney *U* test.

**Table 3:** Characteristics of the subjects (elderly patients and healthy elderly individuals) with postprandial hypotension defined as >20/>30 mmHg systolic fall.

	Patients (*n* = 20/10 out of 22)	Healthy elderly (*n* = 8/3 out of 20)	Range*	*P*
Start (min)	34 ± 27/23 ± 8	34 ± 25/21 ± 13	0–104/10–36	NS/NS
Max fall (mmHg)**	39 ± 19	31 ± 8.3	20–79	NS
Time of max fall (min)**	58 ± 27	48 ± 25	10–110	NS
Duration (min)**	42 ± 36/35 ± 29	28 ± 26/17 ± 22	2–108/2–70	NS/NS
Change in HR (bpm)	8 ± 7	4 ± 5	−11–12	NS
Presence of complaints (*n*)	13/7	4/0		NS/0.01
Start complaints (min)	33 ± 30	36 ± 27	0–84	NS
Duration complaints (min)	55 ± 29	43 ± 32	14–90	NS

Mean ± standard deviation.

*All PPH positives.

**Data about the fall in systolic BP.

*P*: statistical difference between the groups (NS: not significant, when the difference is >.05).

**Table 4:** Test characteristics of “PPH-related symptoms” for the diagnosis PPH. PPH was defined as a fall in systolic blood pressure of >20 or >30 mmHg within 2 hours of the start of the meal, measured with a Portapres device, using all 2-minute samples. The sensitivity of PPH-related symptoms for a fall in blood pressure over 20 mmHg was 17/28 = 0.61, the specificity was 13/14 = 0.93, the positive predictive value was 17/18 = 0.94 and the negative predictive value was 13/24 = 0.54. For a fall in blood pressure over 30 mmHg the sensitivity was 7/13 = 0.53, the specificity was 13/29 = 0.62, the positive predictive value 7/18 = 0.39 and the negative predictive value 18/24 = 0.75.

	PPH	No PPH	Total
PPH-related symptoms present	17/7	1/11	18
PPH-related symptoms absent	11/6	13/18	24

Total	28/13	14/29	42
